# Neural dynamics of verbal working memory in auditory description naming

**DOI:** 10.1038/s41598-018-33776-2

**Published:** 2018-10-26

**Authors:** Toshimune Kambara, Erik C. Brown, Brian H. Silverstein, Yasuo Nakai, Eishi Asano

**Affiliations:** 1 Department of Pediatrics, Children’s Hospital of Michigan, Wayne State University, Detroit, MI 48201 USA; 2 Department of Neurology, Children’s Hospital of Michigan, Wayne State University, Detroit, MI 48201 USA; 3 Translational Neuroscience Program, Wayne State University, Detroit, MI USA; 4 Postdoctoral Fellowship for Research Abroad, Japan Society for the Promotion of Science (JSPS), Chiyoda-ku, Tokyo, 1020083 Japan; 5 Department of Psychology, Hiroshima University, Hiroshima, 7398524 Japan; 6 Department of Neurological Surgery, Oregon Health and Science University, Portland, OR 97239 USA

## Abstract

Auditory naming is suggested to require verbal working memory (WM) operations in addition to speech sound perception during the sentence listening period and semantic/syntactic processing during the subsequent judgement period. We attempted to dissect cortical activations attributable to verbal WM from those otherwise involved in answering auditory sentence questions. We studied 19 patients who underwent electrocorticography recordings and measured high-gamma activity during auditory naming and WM tasks. In the auditory naming task, inferior-precentral high-gamma activity was augmented during sentence listening, and the magnitude of augmentation was independently correlated to that during the WM task maintenance period as well as patient age. High-gamma augmentation during the WM task scanning period accounted for high-gamma variance during the naming task judgement period in some of the left frontal association neocortex regions (most significantly in the middle-frontal, less in the inferior-frontal, and least in the orbitofrontal gyrus). Inferior-frontal high-gamma augmentation was left-hemispheric dominant during naming task judgement but rather symmetric during WM scanning. Left orbitofrontal high-gamma augmentation was evident only during the naming task judgement period but minimal during the WM task scanning period. The inferior-precentral regions may exert WM maintenance during sentence listening, and such maintenance function may be gradually strengthened as the brain matures. The left frontal association neocortex may have a dorsal-to-ventral gradient in functional roles during naming task judgement. Namely, left middle-frontal activation may be well-attributable to WM scanning function, whereas left orbitofrontal activation may be attributable less to WM scanning but more largely to syntactic/semantic processing.

## Introduction

**Figure 1:**
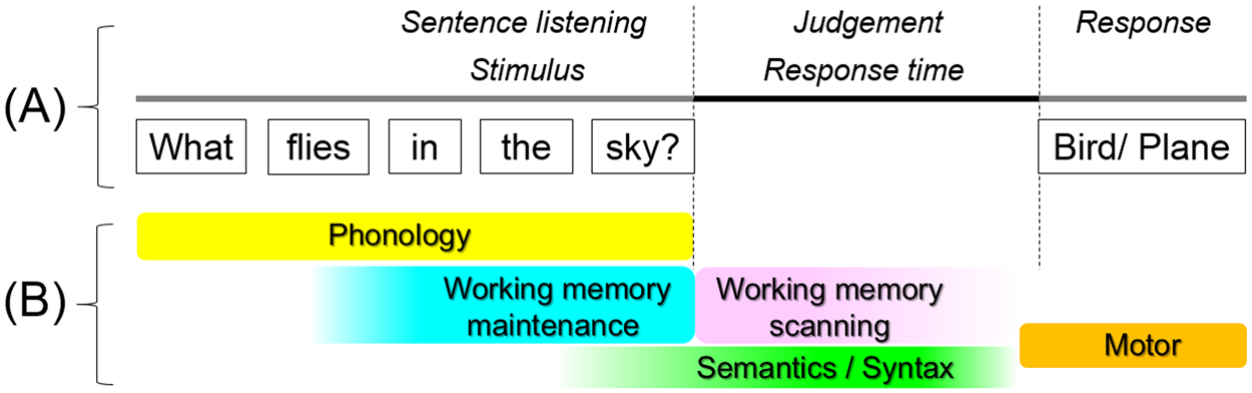
Auditory naming task. (**A**) The task is also known as an auditory description naming task^7,72^. In each trial, a given participant listened to a sentence question and overtly named a relevant answer. The duration of sentence stimuli ranged from 1 to 2.5 s (median: 1.8 s). The response time was defined as the period between stimulus offset and response onset. (**B**) The timing and nature of cerebral functions required to complete the task are hypothesized based on previous literature^1–3^. Phonological processing occurs during stimulus presentation. Simultaneously, working memory maintenance is exerted to maintain a set of words as a single ‘chunk’ for a short time; accordingly, the memory maintenance load is expected to be larger during the latter half of the sentence compared to during the former half^2^. The present study will test the specific hypothesis that inferior-precentral high-gamma augmentation during sentence listening would be at least in part attributable to verbal working memory maintenance operation, by contrasting activation patterns during two different tasks. Semantic/syntactic function is believed to be exerted maximally around question offset and after; simultaneously, working memory scanning is expected to identify a match between internally generated responses and the externally provided question^1,2^. We will determine if cortical activation at a given region after stimulus offset (i.e.: judgement period) would be attributable or non-attributable to this working memory scanning operation.

**Figure 2:**
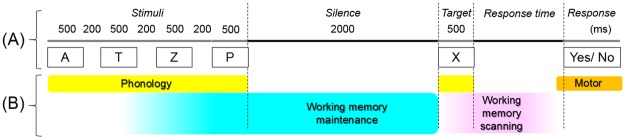
Verbal working memory task. (**A**) The measurement of interest was high-gamma activity during ‘high-load’ working memory trials containing four stimuli. Participants listened to stimulus letters, followed by a 2-s silent period, after which participants heard a target letter and overtly answered ‘Yes’ or ‘No’ regarding whether the target letter was included in the set for a given trial. (**B**) According to previous literature^2,4^, working memory maintenance is theorized to be exerted during a 2-s silent period, whereas working memory scanning subsequently takes place following target onset. Since this working memory task minimally requires semantic or syntactic processing, common and differential high-gamma augmentations during this and the aforementioned task (Fig. 1A) are expected to reveal the profiles of task-related activation that may be attributable to verbal working memory operations. The response time was defined as the period between target offset and response onset.

If you are asked the question: ‘*What flies in the sky?*’ Your answer might be ‘*bird*’ or ‘*plane*’. For comprehension of such a spoken question, humans exert phonological, semantic, and syntactic processing, in conjunction with verbal working memory operations (Fig. 1)^1–3^. Verbal working memory function is suggested to consist of two distinct processes, referred to as (i) *working memory maintenance* characterized by brief storage of mental representations of speech sounds and (ii) *working memory scanning* characterized by subsequent retrieval of what was just heard and for appropriate responses (Fig. 2)^2–4^. Here, we attempted to segregate cortical activation attributable to verbal working memory function from those otherwise involved in semantic and syntactic processing for auditory naming, using measurement of event-related high-gamma modulations on electrocorticography (ECoG)^5,6^. While undergoing extraoperative ECoG recording as part of presurgical evaluation, patients with focal epilepsy were assigned (i) an auditory naming task (i.e.: overt naming in response to a spoken question^7^; Fig. 1) and (ii) an auditory working memory task^8^. This working memory task was designed to effectively localize electrode sites involved in either maintenance or scanning of auditory letter stimuli (Fig. 2). Augmentation of high-gamma activity (70–110 Hz) on ECoG was treated as a summary measure of cortical activation^5,7,9^.

The inferior-precentral gyrus (iPreCG) has been consistently indicated as a key structure in both ECoG and functional magnetic resonance imaging (fMRI) studies using working memory tasks^8,10–15^. Particularly, our previous ECoG study demonstrated that high-gamma augmentation was prominent at iPreCG during the working memory maintenance period, whereas high-gamma augmentation during the working memory scanning period was observed in broad areas including the prefrontal regions^8^. Taking into account that our working memory task does not require semantic or syntactic processing (Fig. 2), we hypothesized that the iPreCG high-gamma augmentation during the sentence listening period of the naming task would be attributed to working memory maintenance function. In the present study, we specifically tested our prediction that the magnitude of high-gamma augmentation at a given iPreCG site during naming task sentence listening (Fig. 1A) would positively correlate to that during working memory task maintenance period (Fig. 2A). We designed the statistical analysis to determine the independent effect of age on high-gamma measures, since patients with a wide range of age were included in the present study.

To clarify the cortical dynamics of naming task judgement process non-attributable to working memory scanning, we determined the spatiotemporal patterns of common and differential high-gamma augmentations during the naming task judgement (i.e.: period after stimulus offset; Fig. 1A) and working memory task scanning periods (Fig. 2A) in the same patient cohort. We specifically predicted that left frontal association neocortices would show greater extent of high-gamma augmentation during the naming task judgement period compared to during the working memory task scanning period.

## Methods

### Participants

The inclusion criteria consisted of patients who underwent the auditory naming (Fig. 1A) and verbal working memory (Fig. 2A) tasks during extraoperative subdural ECoG recording at Children’s Hospital of Michigan or Harper University Hospital. The exclusion criteria consisted of: (i) presence of massive brain malformations, (ii) severe cognitive dysfunction defined by verbal IQ or verbal comprehension index <70, (iii) inability to complete the tasks, (iv) primary language other than English, (v) history of previous epilepsy surgery, and (vi) right-hemispheric language dominance as suggested by the result of Wada test or left-handedness associated with left-hemispheric congenital neocortical lesions (see the rationale in our previous study^7^). Nineteen patients satisfying the inclusion and exclusion criteria were studied (age range: 6–44 years; seven females); this is the identical cohort of patients previously reported in our ECoG study of working memory function (the patient profiles are previously presented^8^). This study was approved by the Institutional Review Board at Wayne State University, and performed in accordance with the approved guidelines. Informed consent was obtained from the patients or guardians of patients.

### Acquisition of ECoG and three-dimensional magnetic resonance surface images

The principal methods of electrophysiology and imaging data acquisition are identical to those previously reported^7,8^. Platinum subdural electrodes (10 mm center-to-center distance; 4 mm diameter; 3 mm exposed diameter) were placed on the affected hemisphere to determine the boundary between the presumed epileptogenic zone to be surgically removed and the eloquent areas to be preserved^16^. Extraoperative ECoG signals were recorded with Nihon Kohden Neurofax 1100 A Digital System (Nihon Kohden America Inc., Foothill Ranch, CA, USA) at a sampling frequency of 1,000 Hz. Electrode sites classified as seizure onset zone as well as those showing interictal spikes or artifacts during either task were excluded from further analysis. The number of analyzed electrodes ranged from 72 to 120 per patient (Table 1).

**Table 1:** The number of electrodes at regions of interest (ROIs).

ROIs	Electrodes (Subjects)	
	Left	Right
STG: superior-temporal gyrus.	120 (14)	45 (6)
MTG: middle-temporal gyrus.	126 (14)	40 (6)
ITG: inferior-temporal gyrus.	78 (13)	27 (6)
FG: fusiform gyrus.	78 (14)	28 (5)
SMG: supra-marginal gyrus.	92 (13)	28 (4)
MFG: middle-frontal gyrus.	119 (13)	56 (6)
IFG: inferior-frontal gyrus.	96 (13)	30 (6)
ORB: orbitofrontal cortex.	74 (13)	23 (6)
iPreCG: inferior-precentral gyrus.	109 (13)	44 (6)
iPoCG: inferior-postcentral gyrus.	91 (13)	36 (6)

The total number of analyzed electrodes (and the number of contributing patients) in each ROI is provided.

**Figure 3:**
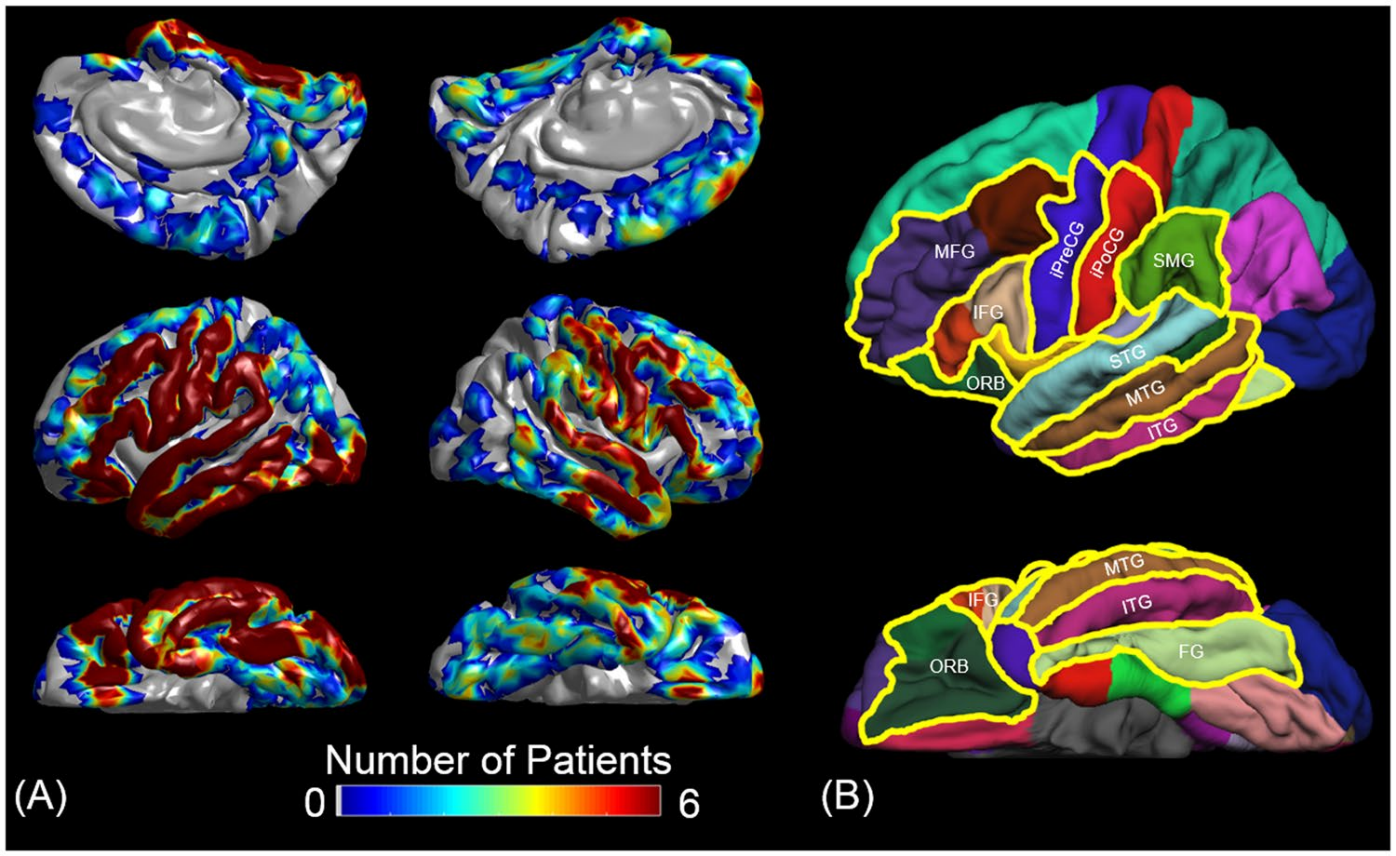
Electrode coverage and regions of interest (ROIs). (**A**) The spatial extent of a total of 1,756 analyzed electrodes are indicated on the FreeSurfer’s average brain images^17^. (**B**) The boundaries of ROIs of the present study are denoted with yellow lines. MFG: middle-frontal gyrus. IFG: inferior-frontal gyrus (summation of pars opercularis [BA 44] and triangularis [BA 45]). ORB: orbitofrontal region (summation of pars orbitalis [BA 47] and lateral-orbitofrontal gyrus). iPreCG: inferior-precentral gyrus. iPoCG: inferior-postcentral gyrus. SMG: supramarginal gyrus. STG, MTG, and ITG: superior-, middle-, and inferior-temporal gyrus, respectively. FG: fusiform gyrus. The number of analyzed electrodes in each ROI was provided in Table 1.

A three-dimensional surface image was created with the location of electrodes directly defined on the brain surface as previously reported^7^. The spatial normalization of individual electrode sites was performed with FreeSurfer scripts (http://surfer.nmr.mgh.harvard.edu). All electrode sites on an individual’s FreeSurfer brain surface were transformed into Talairach coordinates, and finally plotted on the averaged FreeSurfer pial surface image^7,17,18^. Parcellation of cortical gyri was performed at both individual and spatially normalized brain surfaces, and regions of interest (ROIs) analyzed in this study are presented in Fig. 3.

### Auditory naming task

The auditory naming task consisted of question-and-answer trials in which participants were instructed to listen to a sentence question (median duration: 1,800 ms) and to overtly provide a relevant answer^7^ (Fig. 1A). Trials not accompanied by correct noun answers were excluded from further analysis, because patient attentiveness at these particular moments may be in question. For example, when patients were asked ‘*What flies in the sky?*’, incorrect answers would include: *‘I don’t know’, ‘Can you repeat the question?’, ‘What do you mean?’, ‘What flies in the sky?’*, or a gesture response. Conversely, correct answers would include *‘Bird’, ‘Plane’, ‘Superhero’, ‘Dog’*, and so on. Nouns relevant based on each patient’s criteria were treated as correct answers.

The mean number of included trials was 85.4 per patient (standard error [SE]: 2.0). The mean response time was 1,493 ms (SE: 129).

### Verbal working memory task

This task, as described in our previous study^8^ (Fig. 2), represents a letter-based, auditory version on the Sternberg working memory task^4^. Participants were instructed to remember a verbally provided set of two or four letters for 2 s and to overtly decide whether a subsequent target letter had been included. The measurement of interest in the present study was high-gamma activity during ‘high-load’ (four letters) trials accompanied by correct answers alone. Each patient was assigned 30 ‘high-load’ trials, and the mean number of included ‘high-load’ correct-answer trials was 27.6 (SE: 0.7) per patient and the mean response time was 1,383 ms (SE: 190).

### Assessment of ECoG amplitude changes

**Figure 4:**
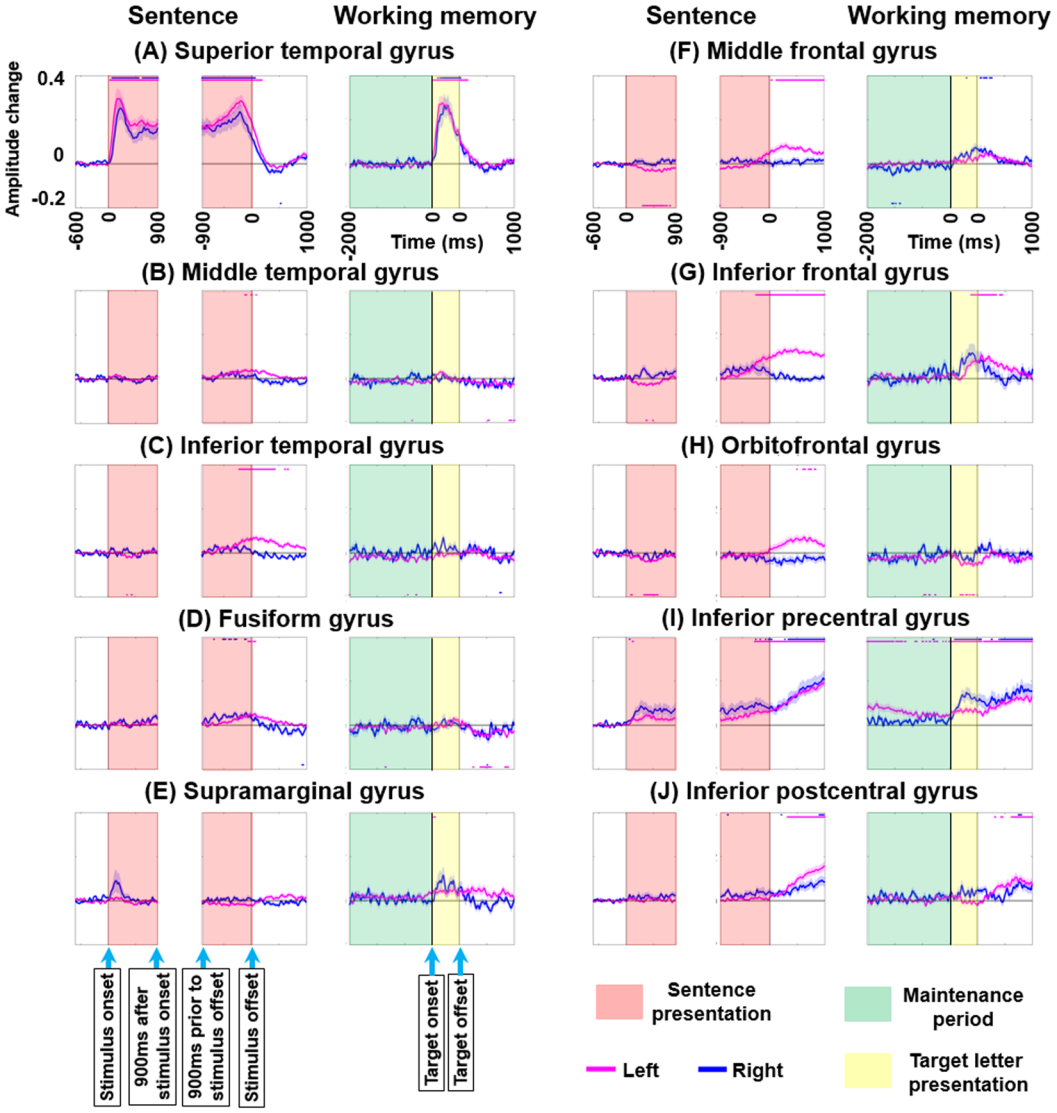
High-gamma dynamics during auditory naming and working memory tasks. Mean high-gamma activity at (**A**) superior-temporal gyrus (STG), (**B**) middle-temporal gyrus (MTG), (**C**) inferior-temporal gyrus (ITG), (**D**) fusiform gyrus (FG), (**E**) supramarginal gyrus (SMG), (**F**) middle-frontal gyrus (MFG), (**G**) inferior-frontal gyrus (IFG), (**H**) orbitofrontal gyrus (ORB), (**I**) inferior-precentral gyrus (iPreCG), and (**J**) inferior-postcentral gyrus (iPoCG). +0.1 indicates 10% increase compared to the baseline value. Red line: left hemisphere. Blue line: right hemisphere. Standard error bars are shown. Horizontal bars above and below: periods with significant high-gamma augmentation and attenuation, respectively. Duration of target letter presentation: 500 ms.

The principal methods are identical to those previously reported^7,8^. A complex demodulation algorithm was employed to transform ECoG signals into the time-frequency domain in steps of 5 Hz and 10 ms^19^. We quantified ‘when’ and ‘where’ high-gamma_70–110 Hz_ amplitudes were modulated by computing the percentage at each 10 ms period relative to a 400-ms reference period during 600–200 ms prior to stimulus onset. A studentized bootstrap analysis, followed by Bonferroni correction for multiple comparisons within a 3,000-ms period (Fig. 4), determined ‘at what moment’ high-gamma amplitude was significantly increased or decreased from the baseline value during the reference period at a given ROI^7,20,21^. The dynamic change of high-gamma amplitude and significance relative to baseline was plotted as a function of time in each ROI during each task (Fig. 4).

### To determine the association between neural activations during naming task sentence listening and working memory task maintenance periods

Using a repeated measures linear mixed model analysis, conducted in SPSS Statistics 24 (IBM Corp., Chicago, IL, USA), we determined whether the variance in the magnitude of high-gamma modulation across individual electrode sites within each ROI during the sentence listening period accounted for high-gamma modulations during working memory maintenance or scanning processes. The dependent variable, derived from the auditory naming task, was [HG _sentence listening_], defined as the mean high-gamma amplitude during the latter half of sentence listening (i.e.: the 900-ms period immediately prior to stimulus offset [Fig. 4]; working memory maintenance load is expected to become greater toward the end of sentence stimuli in general^22^). The following four covariates, including two functional variables derived from the working memory task, were treated as fixed effects: (1) [HG _WM maintenance_]: high-gamma amplitude during the working memory task maintenance period (i.e.: mean across 400-ms period straddling the mid-point of the maintenance period); (2) [HG _WM scanning_]: high-gamma amplitude during the working memory task scanning period, defined as the mean across the 400-ms period between 200 and 600 ms after target offset, which was assumed to be minimally involved in primary auditory or articulation processes taking into account the mean response time^23,24^ (Fig. 2B); (3) [Hemisphere]: 1 if left and 0 if right hemisphere; (4) [Patient age] (years). [Patient] and [Intercept] were treated as random effects. The level of significance was set at p ≤ 0.005, invoking Bonferroni correction for multiple comparisons within 10 regions of interest. Our specific prediction was that [HG _sentence listening_] at an iPreCG site would positively correlate to [HG _WM maintenance_] of the same site. The overall results are presented in Table 2.

**Table 2:** Results of mixed model analysis to determine the association between high-gamma activity during the sentence listening period and those during working memory maintenance/scanning operations. The level of significance was set at p-value at ≤ 0.005, invoking Bonferroni correction for multiple comparisons within 10 regions of interest. HG: high-gamma. WM: working memory.

(A) Superior temporal region	Estimate (95% CI)	t-value	p-value	(F) Middle frontal region	Estimate (95% CI)	t-value	p-value
HG WM maintenance	0.099 (−0.685 to 0.882)	0.249	0.804	HG WM maintenance	0.110 (−0.067 to 0.288)	1.232	0.220
HG WM scanning	−0.649 (−1.239 to −0.059)	−2.176	0.031	HG WM scanning	0.110 (−0.037 to 0.257)	1.472	0.143
Hemisphere	0.022 (−0.099 to 0.143)	0.370	0.714	Hemisphere	−0.008 (−0.059 to 0.042)	−0.352	0.729
Patient age	−0.002 (−0.010 to 0.005)	−0.708	0.487	Patient age	0.001 (−0.002 to 0.004)	0.725	0.479
**(B) Middle temporal region**	**Estimate (95% CI)**	**t-value**	**p-value**	**(G) Inferior frontal region**	**Estimate (95% CI)**	**t-value**	**p-value**
HG WM maintenance	0.153 (−0.082 to 0.387)	1.287	0.200	HG WM maintenance	0.072 (−0.287 to 0.431)	0.397	0.692
HG WM scanning	0.067 (−0.139 to 0.272)	0.643	0.521	HG WM scanning	0.049 (−0.193 to 0.290)	0.399	0.690
Hemisphere	0.020 (−0.022 to 0.061)	0.948	0.348	Hemisphere	0.014 (−0.056 to 0.083)	0.405	0.689
Patient age	0.001 (−0.001 to 0.003)	0.765	0.453	Patient age	0.001 (−0.002 to 0.005)	0.765	0.454
**(C) Inferior temporal region**	**Estimate (95% CI)**	**t-value**	**p-value**	**(H) Orbitofrontal region**	**Estimate (95% CI)**	**t-value**	**p-value**
HG WM maintenance	0.637 (0.362 to 0.913)	4.586	0.000	HG WM maintenance	−0.061 (−0.331 to 0.208)	−0.453	0.652
HG WM scanning	−0.272 (−0.454 to −0.090)	−2.961	0.004	HG WM scanning	0.055 (−0.175 to 0.285)	0.474	0.636
Hemisphere	0.031 (−0.015 to 0.077)	1.353	0.184	Hemisphere	0.012 (−0.039 to 0.063)	0.461	0.647
Patient age	0.001 (−0.001 to 0.004)	0.924	0.365	Patient age	0.000 (−0.004 to 0.003)	−0.214	0.833
**(D) Fusiform region**	**Estimate (95% CI)**	**t-value**	**p-value**	**(I) Inferior precentral region**	**Estimate (95% CI)**	**t-value**	**p-value**
HG WM maintenance	−0.083 (−0.282 to 0.115)	−0.832	0.407	HG WM maintenance	0.293 (0.097 to 0.488)	2.952	0.004
HG WM scanning	0.044 (−0.144 to 0.232)	0.462	0.645	HG WM scanning	−0.086 (−0.260 to 0.088)	−0.983	0.328
Hemisphere	−0.017 (−0.059 to 0.026)	−0.803	0.430	Hemisphere	0.015 (−0.061 to 0.091)	0.416	0.682
Patient age	0.000 (−0.002 to 0.002)	0.271	0.790	Patient age	0.006 (0.002 to 0.010)	3.133	0.005
**(E) Supramarginal region**	**Estimate (95% CI)**	**t-value**	**p-value**	**(J) Inferior postcentral region**	**Estimate (95% CI)**	**t-value**	**p-value**
HG WM maintenance	0.023 (−0.145 to 0.191)	0.272	0.786	HG WM maintenance	0.100 (−0.035 to 0.234)	1.467	0.145
HG WM scanning	−0.009 (−0.126 to 0.109)	−0.144	0.886	HG WM scanning	−0.024 (−0.114 to 0.067)	−0.518	0.605
Hemisphere	−0.001 (−0.043 to 0.040)	−0.071	0.944	Hemisphere	0.007 (−0.033 to 0.048)	0.408	0.691
Patient age	0.001 (−0.001 to 0.003)	1.082	0.294	Patient age	0.003 (0.000 to 0.005)	2.618	0.022

### To determine the association between neural activations during naming task judgement and working memory task scanning periods

Likewise, we determined whether the variance in the magnitude of left-hemispheric high-gamma modulation during the naming task judgement period accounted for high-gamma modulations during working memory maintenance or scanning processes. The dependent variable, again computed from the auditory naming task, [HG _naming task judgement_], was defined as the mean high-gamma amplitude during a 400-ms period between 200 and 600 ms after sentence offset. This 400-ms period was assumed to be minimally involved by primary auditory or articulation process taking into account the mean response time (Fig. 1B). The fixed and random predictors were the same as stated above. Our specific prediction was that [HG _naming task judgement_] would positively correlate to [HG _WM scanning_] within some but not all of the analyzed ROIs. Bonferroni correction was likewise employed. The overall results are presented in Table 3.

**Table 3:** Results of mixed model analysis to determine the association between high-gamma activity during the sentence judgement period and those during working memory maintenance/scanning operations. The level of significance was set at p-value at ≤ 0.005, invoking Bonferroni correction for multiple comparisons within 10 regions of interest. HG: high-gamma. WM: working memory.

(A) Superior temporal region	Estimate (95% CI)	t-value	p-value	(F) Middle frontal region	Estimate (95% CI)	t-value	p-value
HG WM maintenance	0.181 (−0.042 to 0.403)	1.604	0.111	HG WM maintenance	0.006 (−0.256 to 0.267)	0.043	0.966
HG WM scanning	0.280 (0.113 to 0.448)	3.314	0.001	HG WM scanning	0.482 (0.265 to 0.699)	4.389	0.000
Hemisphere	0.016 (−0.019 to 0.052)	0.970	0.348	Hemisphere	0.071 (−0.004 to 0.146)	1.988	0.063
Patient age	0.000 (−0.002 to 0.002)	−0.249	0.808	Patient age	0.001 (−0.003 to 0.005)	0.559	0.584
**(B) Middle temporal region**	**Estimate (95% CI)**	**t-value**	**p-value**	**(G) Inferior frontal region**	**Estimate (95% CI)**	**t-value**	**p-value**
HG WM maintenance	0.074 (−0.169 to 0.317)	0.604	0.547	HG WM maintenance	0.155 (−0.203 to 0.513)	0.858	0.393
HG WM scanning	0.393 (0.180 to 0.607)	3.635	0.000	HG WM scanning	0.262 (0.022 to 0.503)	2.158	0.033
Hemisphere	0.030 (−0.015 to 0.075)	1.335	0.188	Hemisphere	0.105 (0.039 to 0.172)	3.232	0.003
Patient age	0.000 (−0.003 to 0.002)	−0.198	0.845	Patient age	0.000 (−0.003 to 0.004)	0.186	0.855
**(C) Inferior temporal region**	**Estimate (95% CI)**	**t-value**	**p-value**	**(H) Orbitofrontal region**	**Estimate (95% CI)**	**t-value**	**p-value**
HG WM maintenance	0.242 (−0.066 to 0.550)	1.558	0.123	HG WM maintenance	−0.135 (−0.542 to 0.271)	−0.662	0.510
HG WM scanning	0.316 (0.112 to 0.519)	3.082	0.003	HG WM scanning	0.407 (0.065 to 0.750)	2.360	0.020
Hemisphere	0.056 (0.000 to 0.113)	2.011	0.051	Hemisphere	0.059 (−0.022 to 0.140)	1.468	0.149
Patient age	−0.001 (−0.004 to 0.002)	−0.761	0.455	Patient age	−0.004 (−0.009 to 0.002)	−1.318	0.206
**(D) Fusiform region**	**Estimate (95% CI)**	**t-value**	**p-value**	**(I) Inferior precentral region**	**Estimate (95% CI)**	**t-value**	**p-value**
HG WM maintenance	−0.103 (−0.357 to 0.152)	−0.801	0.425	HG WM maintenance	0.178 (0.049 to 0.307)	2.732	0.007
HG WM scanning	0.264 (0.021 to 0.506)	2.158	0.033	HG WM scanning	0.622 (0.503 to 0.740)	10.365	0.000
Hemisphere	0.021 (−0.044 to 0.086)	0.665	0.512	Hemisphere	0.037 (−0.039 to 0.113)	1.041	0.315
Patient age	0.000 (−0.004 to 0.003)	−0.066	0.948	Patient age	0.003 (−0.001 to 0.007)	1.501	0.155
**(E) Supramarginal region**	**Estimate (95% CI)**	**t-value**	**p-value**	**(J) Inferior postcentral region**	**Estimate (95% CI)**	**t-value**	**p-value**
HG WM maintenance	0.029 (−0.212 to 0.270)	0.238	0.812	HG WM maintenance	0.201 (0.016 to 0.386)	2.155	0.033
HG WM scanning	0.250 (0.082 to 0.418)	2.945	0.004	HG WM scanning	0.751 (0.624 to 0.878)	11.699	0.000
Hemisphere	0.028 (−0.031 to 0.087)	1.006	0.329	Hemisphere	0.027 (−0.039 to 0.094)	0.867	0.398
Patient age	0.001 (−0.002 to 0.004)	0.824	0.420	Patient age	0.003 (−0.001 to 0.006)	1.560	0.136

## Results

### iPreCG high-gamma augmentation during naming task sentence listening correlated to high-gamma during working memory task maintenance period

**Figure 5:**
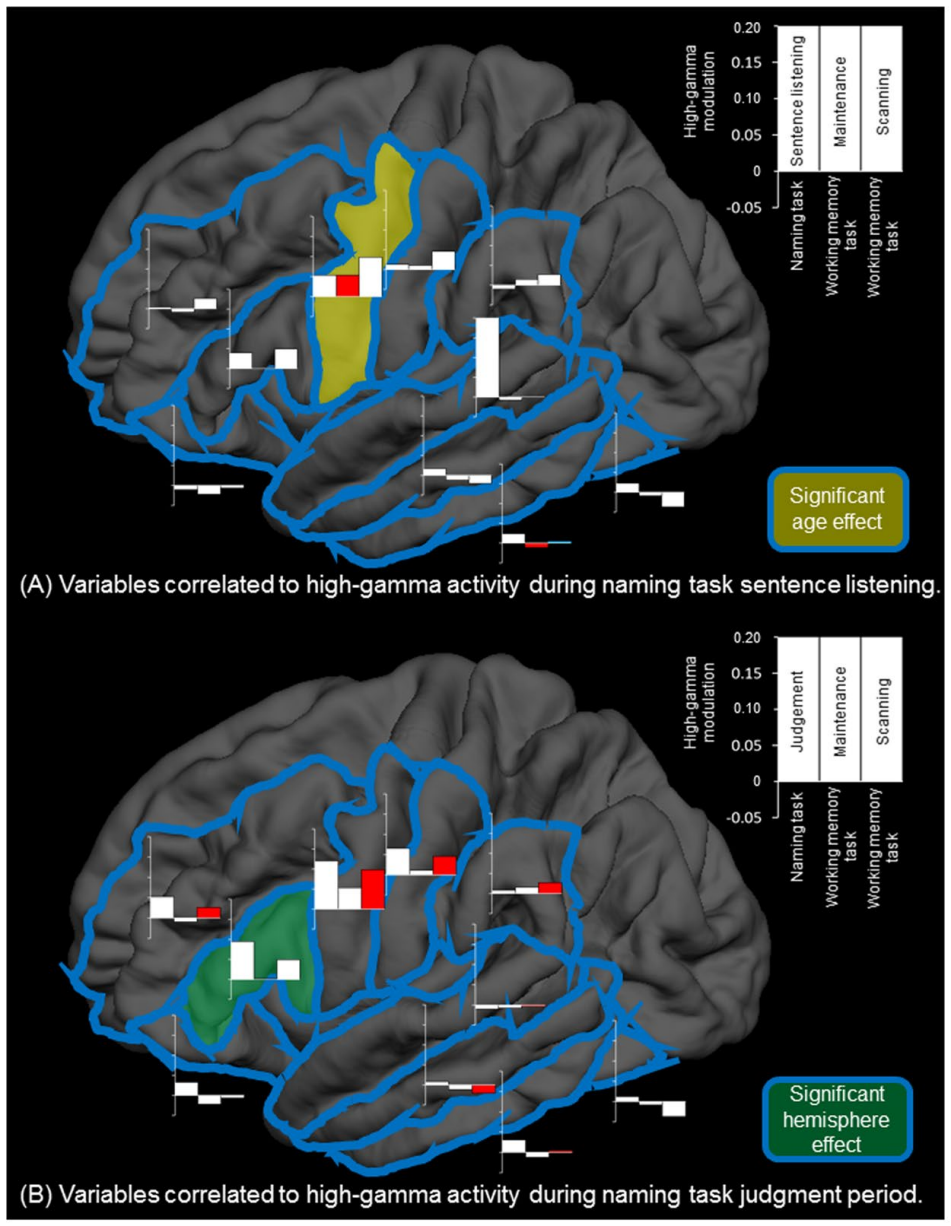
Association between high-gamma activities during naming and working memory tasks. (**A**) Variables correlated to high-gamma activity during naming task sentence listening are presented. Bar graphs at each region of interest show the degree of high-gamma modulation during naming task sentence listening period (left), working memory task maintenance period (center), and working memory task scanning period (right). +0.10 indicates 10% increase compared to the baseline value. Red and blue bars respectively indicate positive and negative correlation between high-gamma activity during sentence listening period and that during working memory task maintenance or scanning period, based on the results of mixed model analysis. We found that iPreCG high-gamma activity during sentence listening was positively correlated to that during working memory task maintenance period as well as patient age. (**B**) Variables correlated to high-gamma activity during naming task judgement period are presented. We found that high-gamma activity during naming task judgment period was positively correlated to that during working memory task scanning period at multiple regions of interest but not at IFG or ORB. IFG high-gamma activity during naming task judgement period was significantly larger in the left compared to the right. Left middle-temporal high-gamma activity during working memory task scanning period was rather attenuated compared to that during the baseline period; thereby, electrode sites showing larger high-gamma attenuation were associated with smaller high-gamma augmentation during naming task judgement.

Figure 5A summarizes the association between high-gamma activity during naming task sentence listening and during both working memory task maintenance and scanning periods. In the working memory task, high-gamma augmentation during the maintenance period reached significance in the iPreCG (Fig. 4I) but not in the remaining ROIs. In the auditory naming task, high-gamma augmentation was noted at iPreCG during sentence listening period (Fig. 4I). Mixed model analysis demonstrated that [HG _sentence listening_] positively correlated with [HG _WM maintenance_] (estimate of mixed model regression coefficient = +0.293 [95%CI: + 0.097 to + 0.488]; p = 0.004) and [Patient age] (estimate = +0.006 [95%CI: + 0.002 to + 0.010]; p = 0.005) but not with [HG _WM scanning_] (estimate = −0.086 [95%CI: −0.260 to +0.080]; p = 0.328). [Hemisphere] had no effect on [HG _sentence listening_]. In other words, each 1-year increment in age resulted in a 0.6% increase in the degree of iPreCG high-gamma augmentation during the sentence listening period of the naming task.

### iPreCG and iPoCG high-gamma augmentation peaks toward response onset

Following stimulus offset during the auditory naming task as well as target offset during the working memory task, iPreCG and inferior-postcentral gyrus (iPoCG) showed significant high-gamma augmentation, bilaterally (Fig. 4I and J). High-gamma augmentation at iPreCG preceded that at iPoCG, and the intensity of iPreCG and iPoCG high-gamma augmentation gradually increased toward the onset of response in both tasks. Mixed model analysis demonstrated that [HG _naming task judgement_] tightly correlated to [HG _WM scanning_] at iPreCG (estimate = +0.622 [95%CI: + 0.503 to + 0.740]; p < 0.001) as well as at iPoCG (estimate = +0.751 [95%CI: + 0.624 to + 0.878]; p < 0.001), whereas neither [HG _WM maintenance_], [Hemisphere], nor [Patient age] had a significant effect on [HG _naming task judgement_].

### High-gamma augmentation in the other frontal regions during naming task judgement and working memory task scanning periods

Figure 5B summarizes the association between high-gamma activity during naming task judgement period and those during working memory task maintenance and scanning periods. Following stimulus offset during the auditory naming task, high-gamma augmentation was noted at the inferior-frontal gyrus (IFG), middle-frontal gyrus (MFG), and orbitofrontal gyrus (ORB) of the left but not of the right hemisphere (Fig. 4F,G and H). Mixed model analysis demonstrated a significant effect of [Hemisphere] on [HG _naming task judgement_] at IFG (estimate = +0.105 [95%CI: + 0.039 to + 0.172]; p = 0.003), whereas the effects of [Hemisphere] at the other two ROIs failed to reach significance.

Following target offset during the working memory task, high-gamma activity was bilaterally augmented at IFG and MFG (Fig. 4F and G). Conversely, high-gamma augmentation was minimal at ORB during the same time period (Fig. 4H); instead, left ORB high-gamma augmentation was specific to the auditory naming task. Mixed model analysis demonstrated that [HG _naming task judgement_] at MFG correlated to [HG _WM scanning_] (estimate = +0.482 [95%CI: + 0.265 to + 0.699]; p < 0.001) but not to [HG _WM maintenance_] (estimate = +0.006 [95%CI: −0.256 to + 0.267]; p = 0.966). On the other hand, [HG _naming task judgement_] at IFG or ORB did not correlate to [HG _WM scanning_] or [HG _WM maintenance_] with statistical significance.

### Temporal lobe high-gamma augmentation during naming task judgment and working memory task scanning periods

Presentation of vocal sounds, including sentence questions in the auditory naming task and letters in the working memory task, elicited high-gamma augmentation in the superior-temporal gyrus (STG) of either hemisphere (Fig. 4A).

Around and following stimulus offset of the auditory naming task, high-gamma augmentation was noted at inferior-temporal gyrus (ITG), middle-temporal gyrus (MTG), and fusiform gyrus (FG) of the left but not of the right hemisphere (Fig. 4B,C and D). During the scanning period of the working memory task, high-gamma activity was minimally augmented at ITG and MTG (Fig. 4B and C), and left FG high-gamma activity was rather attenuated (Fig. 4D). Mixed model analysis demonstrated that correlation between [HG _naming task judgment_] and [HG _WM scanning_] was strongest at MTG (estimate = +0.393 [+0.180 to +0.607]; p < 0.001), weaker at ITG (estimate = +0.316 [+0.112 to +0.519]; p = 0.003), and further weaker at FG (estimate = +0.264 [+0.021 to +0.506]; p = 0.033).

## Discussion

### Significance of inferior-precentral high-gamma augmentation during naming task sentence listening

The present study supported our hypothesis that iPreCG contributes to verbal working memory maintenance during listening to auditory sentence questions. iPreCG high-gamma augmentation reached significance at the near end of sentence listening when the memory load increased (Fig. 4I). Mixed model analysis demonstrated that iPreCG high-gamma activity during sentence listening correlated to high-gamma activity during the working memory task maintenance period but not during the scanning period. Our hypothesis is also consistent with the ‘phonological loop model’, suggesting that accurate vocal responses to speech sounds are secured by “short-term maintenance of stimuli via sub-vocal rehearsal”^3,25,26^. Convergent with the present data, previous studies using cortico-cortical evoked potentials suggest that STG is directly and functionally connected to iPreCG of the same hemisphere^27,28^. Data from MRI tractography suggest that STG and iPreCG are connected mainly via the arcuate and uncinate fasciculi^29–33^, which are thus the most likely candidates for the conduction of information through this portion of the phonological loop. Further studies using multimodal tools are warranted to determine the exact nature of the dynamic neural information flow between the STG and iPreCG.

Our ECoG studies including patients with a wide range of age allowed us to determine the effect of age on neural activation during the auditory naming task. A positive correlation between iPreCG high-gamma augmentation during sentence listening and patient age is consistent with the notion that more mature individuals are capable of understanding more lengthy or dense instructions with strengthening of working memory maintenance from preschool to young adulthood^34–36^. Previous fMRI studies also reported age-dependent increase in hemodynamic activation during working memory tasks in the frontal lobe structures including the iPreCG^37,38^.

The present study by no means indicates that speech perceptual processing alone contributes to iPreCG high-gamma augmentation during sentence listening. Previous neuroimaging and neurophysiological studies have implicated iPreCG and IFG in the processing of speech perception^39–43^. Yet, we do not have definitive evidence that iPreCG high-gamma augmentation can be attributed to a perceptual processing alone. Our previous ECoG study of 100 patients showed that high-gamma augmentation at STG during sentence listening is initially greater and subsequently declined as a function of time, whereas high-gamma augmentation at iPreCG is initially modest but enhanced as memory load is presumably increasing over time^7^; furthermore, the onset of high-gamma augmentation at iPreCG during sentence listening was delayed by 80–100 ms compared that at STG.

### Significance of inferior precentral and postcentral high-gamma augmentation prior to the onset of overt response

During both naming and working memory tasks, high-gamma augmentation at bilateral iPreCG and iPoCG increased toward response onset (Fig. 4I and J). Our interpretation is that such high-gamma augmentation partly reflects preparation and execution of overt motor responses in addition to likely somatosensory activation. Supporting this interpretation, previous studies using electrical stimulation suggest that both iPreCG and iPoCG have critical face/throat motor function^7,44^. Previous ECoG studies reported that reading of visually-presented sentences elicited high-gamma augmentation in the left iPreCG^45,46^.

In the auditory naming task, high-gamma activity at iPreCG began to be increased even prior to sentence stimulus offset and again increased until the overt response, during which it continued in a sustained manner (Fig. 4I). Mixed model analysis demonstrated that iPreCG high-gamma activity during naming task sentence listening correlated to that during the working memory task maintenance period, whereas iPreCG high-gamma activity during naming task judgment correlated to that during working memory task scanning period. Previous lesion and fMRI studies have indicated that the cortical network supporting working memory maintenance and scanning likely includes the left inferior-parietal and inferior-frontal gyri^47^. The collective evidence led to the hypothesis that iPreCG comprises a part of multiple large-scale networks responsible for working memory maintenance, subsequent scanning, as well as overt articulation during auditory naming task. Future studies incorporating cortical mapping using electrical stimulation time-locked to each period of interest, as well as analysis of ECoG high-gamma information flow, such as event-related causality analysis^28,48^, may provide important convergent evidence for this hypothesis.

### Significance of hemispheric lateralization of high-gamma modulation during naming task judgement

Figure 4 indicates that high-gamma augmentation during the naming task judgement period (i.e.: following sentence stimulus offset) was largely left-hemisphere dominant in frontal and temporal ROIs, in contrast to high-gamma activity during working memory task scanning period (i.e.: following target offset), which tended to be symmetric. Our mixed-model analysis indeed demonstrated that IFG high-gamma activity during naming the task judgement period was left-hemisphere dominant with statistical significance. However, the exact mechanism of left-hemisphere dominant high-gamma augmentation specific to naming task judgement is unknown. In support of the role of the left hemisphere bias in semantic processing, previous fMRI studies of healthy individuals have consistently reported left-hemisphere dominant hemodynamic activation during a covert word finding task requiring semantic processing, as well as age-progressive left-hemispheric dominance, reflecting more efficient language skills in adults relative to children^49–51^. Our previous ECoG study of 100 patients demonstrated that functional inhibition of the right ORB preceded activation of the homotopic region of the left hemisphere during the auditory naming task^7^.

Conversely, the degree of high-gamma augmentation at MFG/IFG during the working memory task scanning period was relatively symmetric between hemispheres (Fig. 4F and G). This observation is consistent with the notion that right MFG/IFG function exerted during the working memory scanning may be unnecessary for judgment during the naming task. An alternative interpretation is that working memory scanning function is distributed across hemispheres, perhaps predominantly so for nonverbal items^47,52–55^. Previous functional imaging studies have demonstrated grossly symmetric or even right-hemispheric dominant MFG/IFG activation elicited by tasks that require working memory of spectral features of sound, temporal order, location, or nonverbal items^52–55^. Given that the working memory task used in the present study can be executed without semantic processing, but rather by simply accurately detecting the difference in spectral features of speech stimuli, these fMRI findings may explain the observed right-hemispheric high-gamma augmentation during the working memory task scanning period.

### Significance of frontal lobe high-gamma augmentation during naming task judgement

Left IFG/MFG showed high-gamma augmentation during naming task judgement as well as during working memory task scanning periods (Fig. 4F and G). This ECoG observation is consistent with the notion that left IFG/MFG high-gamma augmentation following sentence offset, at least in part, reflects verbal working memory scanning. Indeed, our mixed model analysis demonstrated that MFG high-gamma activity during naming task judgement correlated to that during working memory task scanning period. Previous lesion studies, as well as those using functional imaging methodologies, suggest that left IFG and MFG are crucially involved in verbal working memory operations^56–61^. While functional imaging studies do not have sufficient temporal resolution to distinguish activations related to working memory scanning from that related to maintenance, the high temporal resolution of ECoG allowed us to observe separate patterns for these two phases.

Conversely, left ORB (Fig. 3) showed significant high-gamma augmentation specifically during naming task judgement and not during working memory scanning (Fig. 4H). This ECoG finding supports the notion that left ORB high-gamma augmentation during naming task judgement is largely non-attributable to verbal working memory operations. Existing literature suggests the left ORB may be involved in both semantic and syntactic functions. However, with the current tasks, we were not able to differentiate activity in the left ORB related to semantic versus syntactic processing. Functional neuroimaging studies have been able to shed some light on this differentiations: an fMRI study of eight healthy adults using auditory-delivered sentence stimuli reported that the left pars orbitalis region (BA 47; a part of ORB) was selectively involved in the semantic aspect of a sentence, whereas left pars opercularis (BA 44; a part of IFG) was involved in syntactic processing^62^. Furthermore, in a meta-analysis of functional imaging studies, investigators proposed that left ORB/IFG has a functional gradient in which left IFG exerts more semantic processing, whereas left ORB does syntactic^63,64^.

### Significance of temporal lobe high-gamma augmentation during naming task judgement

Around and following stimulus offset during the auditory naming task, high-gamma augmentation was noted most prominently at left ITG, with modest augmentations at MTG and FG (Fig. 4B,C and D), whereas high-gamma activity was minimally augmented or rather attenuated in these regions during the working memory task scanning period. These findings are consistent with the notion that high-gamma augmentation in these temporal lobe structures during naming task judgement includes neural activation non-attributable to working memory scanning. This activation is more likely associated with the production of words semantically relevant to a given sentence question. This role for the left ITG, MTG, and FG is supported by a number of fMRI and lesion studies which have suggested that left ITG, MTG, and also FG are involved in semantic processing^65–71^.

### Limitations of the study

We cannot rule out the possibility that high-gamma augmentation at a cortical site during two tasks is attributable to the exactly same underlying computation. It is possible that neurons at a recording site may be engaged in two entirely different cognitive/sensorimotor processes during two different tasks. For example, in theory, an iPreCG site might be engaged in working memory maintenance function during a task and in another function during a different task.

The number of eligible electrodes in each ROI was controlled between the auditory naming and working memory tasks, allowing for the same statistical power between tasks. However, the number of eligible electrodes differed across ROIs (Table 1); for example, 126 electrodes were available for analysis in left MTG whereas 74 were available in left ORB. Thus, the statistical power of left MTG was approximately 1.3 times greater than that of left ORB in each task (Fig. 4). Furthermore, the number of trials was greater in the auditory naming task compared to the working memory task. The difference in trial numbers between the tasks is reflected by a difference in the signal-to-noise ratios. This effect can be observed in Fig. 4, in which readers might find that high-gamma signal deflections appear somewhat noisier in the working memory task. Based on this, a lack of significant high-gamma augmentation or attenuation in some ROIs during the working memory task should be treated as failure to reach significance possibly due to a low signal-to-noise ratio that may have contributed to reducing the power to find a difference. Our observation of a significant hemispheric effect on the IFG high-gamma activity during naming task judgement is difficult to attribute merely to the effect of imbalance in electrode numbers across hemispheres. The standard error of IFG high-gamma activity during this period was equally small in both hemispheres (1.5% in the left and 1.2% in the right; Fig. 4G). Our study did not have a sufficient statistical power to determine the effect of other covariates (e.g.: nature of MRI lesion) on the degree of high-gamma augmentation during auditory naming task.
